# The Development of Functional Non-Viral Vectors for Gene Delivery

**DOI:** 10.3390/ijms20215491

**Published:** 2019-11-04

**Authors:** Suryaji Patil, Yong-Guang Gao, Xiao Lin, Yu Li, Kai Dang, Ye Tian, Wen-Juan Zhang, Shan-Feng Jiang, Abdul Qadir, Ai-Rong Qian

**Affiliations:** 1 Lab for Bone Metabolism, Key Lab for Space Biosciences and Biotechnology, School of Life Sciences, Northwestern Polytechnical University, Xi’an 710072, China; psuryaji@gmail.com (S.P.); linxiao@nwpu.edu.cn (X.L.); liyu@nwpu.edu.cn (Y.L.); dangkai@nwpu.edu.cn (K.D.); wenjuan_zhang2017@nwpu.edu.cn (W.-J.Z.); abdulqadirwazir145@yahoo.com (A.Q.); 2 Research Center for Special Medicine and Health Systems Engineering, School of Life Sciences, Northwestern Polytechnical University Xi’an 710072, China; 3 NPU-UAB Joint Laboratory for Bone Metabolism, School of Life Sciences, Northwestern Polytechnical University, Xi’an 710072, China

**Keywords:** gene therapy, functional non-viral vectors, fluorescent imaging, biodegradation, targeted delivery

## Abstract

Gene therapy is manipulation in/of gene expression in specific cells/tissue to treat diseases. This manipulation is carried out by introducing exogenous nucleic acids, such as DNA or RNA, into the cell. Because of their negative charge and considerable larger size, the delivery of these molecules, in general, should be mediated by gene vectors. Non-viral vectors, as promising delivery systems, have received considerable attention due to their low cytotoxicity and non-immunogenicity. As research continued, more and more functional non-viral vectors have emerged. They not only have the ability to deliver a gene into the cells but also have other functions, such as the performance of fluorescence imaging, which aids in monitoring their progress, targeted delivery, and biodegradation. Recently, many reviews related to non-viral vectors, such as polymers and cationic lipids, have been reported. However, there are few reviews regarding functional non-viral vectors. This review summarizes the common functional non-viral vectors developed in the last ten years and their potential applications in the future. The transfection efficiency and the transport mechanism of these materials were also discussed in detail. We hope that this review can help researchers design more new high-efficiency and low-toxicity multifunctional non-viral vectors, and further accelerate the progress of gene therapy.

## 1. Introduction

Gene therapy is a promising strategy for the treatment of diseases, such as inherited disorders, viral infections, and cancers. A major limitation of clinical gene therapy is the lack of safe and efficient gene carriers [1]. Over the past few decades, due to advances in molecular biology combined with high-throughput screening techniques, the identification of countless genomic targets of various genetic and acquired disorders, such as cystic fibrosis, cancer, and AIDS, etc., has become possible. Large interest has been orientated toward treating diseases by introducing nucleic acids into the specific cell to target a particular gene responsible for a disease. Therefore, it might hold great potential for the diagnosis and treatment of a variety of diseases, such as hemophilia, cystic fibrosis cancer, and AIDS. The next generation of disease-modifying interventions has been become possible due to the therapeutic and promising effects accomplished by gene therapy [2,3]. For gene therapy, on the one hand, the gene must be suitably delivered to the cells at the targeted site [4]; on the other hand, gene carriers should encase and protect nucleic acids from being rapidly degraded by serum nucleic acid degrading enzymes present in the blood. Therefore, it remains a major challenge to develop an effective and safe carrier for gene delivery [5].

**Figure 1:**
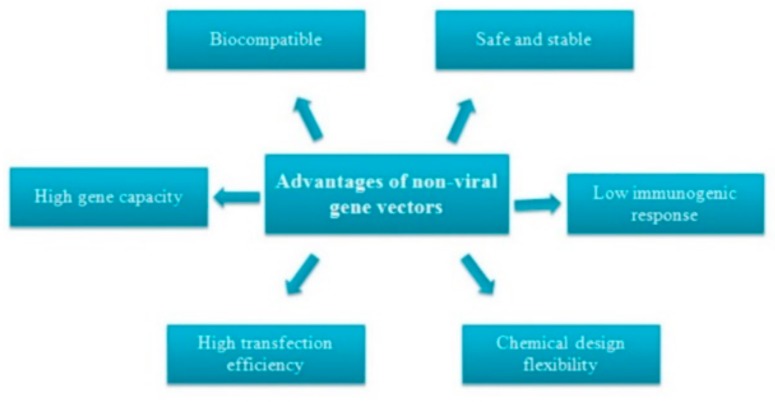
Advantages of non-viral vectors.

Recently viral vectors and non-viral vectors have been used as gene carriers for effective and safe gene delivery. Due to the intracellular gene delivery pathway, viral vectors have the advantage of high gene transfection. However, their use is limited due to side effects (Table 1). Therefore, non-viral vectors with advantages (Figure 1), such as low immunogenic response, safety, high gene capacity, and stability and chemical design flexibility, have received increasing attention. Furthermore, it is practically easy to create and chemically alter non-viral vectors on a large scale. Most importantly, the non-viral vector system is not restricted by the molecular size of the gene to be introduced [6,7,8]. 

Because of these advantages, non-viral vectors, such as polymers [3,10,11,12], lipids [13,14,15,16], and functional vectors [2,5,6], have been developed and applied in gene therapy. Although there are already many review articles that discuss non-viral vectors, such as cationic polymers and cationic lipids, they mainly focus on the structure–activity relationship of these materials or the biological barriers to gene delivery, such as poly(*β*-amino esters) (PBAEs) linear polymers [17], hydrophobic chains modified cationic lipids [18], headgroup evolution of cationic lipids [19], surface-engineered dendrimers [1], morphologically distinct cationic lipid–nucleic acid carriers [20], etc. However, the functional non-viral vectors have seldom been discussed comprehensively and systematically.

In this article, we summarize the recent advancements made in the functional non-viral vectors for the delivery of nucleic acids and discuss the future potential and development of non-viral vectors for gene delivery. Here, we divide functional vectors into four categories: functional fluorescent and non-fluorescent vectors, targeting vectors, biodegradable vectors, and other functional vectors. Functional fluorescent and non-fluorescent vectors include organic fluorescent vectors, quantum dots [21,22,23,24], metal complexes [5,6,25,26,27,28], and fluorescent nanoparticles [29,30,31,32], etc.

## 2. Functional Fluorescent and Non-Fluorescent Vectors

### 2.1. Organic Fluorescent Vectors

#### 2.1.1. Organic Fluorescent Lipids

**Figure 2:**
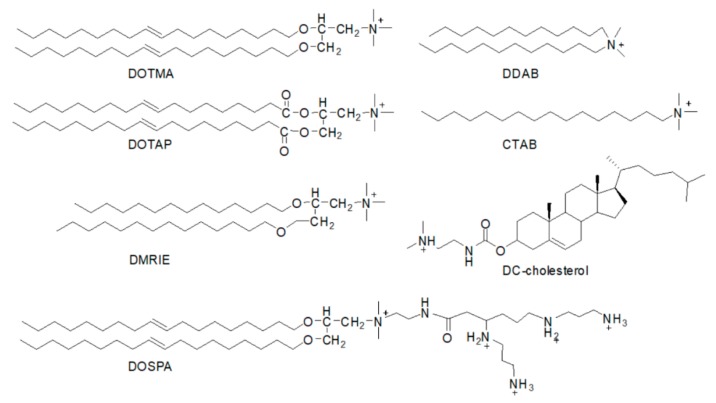
The structures of cationic lipids.

Lipids are a diverse set of compounds with the defining feature of insolubility in water. Biological membranes contain phospholipids and sterols as major structural elements. This fact has been utilized in the preparation of non-viral vectors as gene carries to facilitate an easy delivery [13]. Since the first cationic lipid DOTMA was prepared in 1987, a lot of similar cationic lipids, such as DOTAP, DDAB, and CTAB, have been prepared and applied in gene delivery in different cell types (Figure 2) [33,34,35,36].

Cationic lipids, being important and potential non-viral gene vectors, have more advantages, such as biodegradability, low cytotoxicity, structure variety, and easy production, as compared to other systems. Despite their low immunogenicity, non-toxicity, and easy synthesis, cationic lipids have low transfection efficiency. The most common methods used to improve transfection efficiency include modification of the hydrophobic domains, linker domains, and hydrophilic domains of the lipid and addition of other moieties to the lipids.

**Figure 3:**
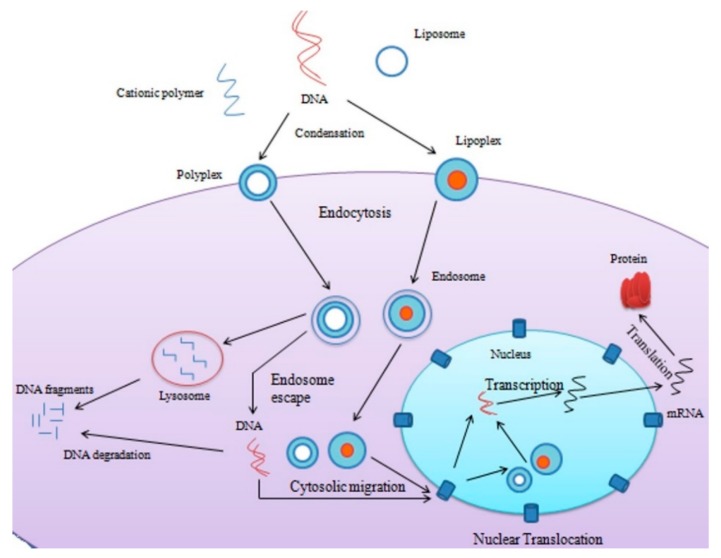
Basic mechanism of non-viral gene delivery via polyplex and lipoplex.

The hydrophobic modification is an effective approach to enhance endosomal escape and improve gene transfection abilities [16,37]. The transport process can be categorized into five steps: the extra-cellular gene delivery, cellular uptake, endosomal escape, nuclear translocation, and transcription/translation (Figure 3) [14]. Therefore, the molecular platforms have allowed producing cationic lipids with additional properties, such as fluorescence, stealth, targeting, etc., without affecting the amphiphilic nature of the lipids [38]. And because of these advancements, the organic fluorescent lipids have achieved advantages, such as simplicity of synthesis, biodegradability, cellular monitoring, and good repeatability, over other vectors [37]. The distinct elements of the cationic lipids, such as the hydrophobic domain, the cationic head group, serves as a scaffold for the other domains, while linkers between the backbone, hydrophobic domain, and cationic head group, the polyethylene glycol chains, serve as the backbone used in gene therapy [36].

Another modification to improve the transfection efficiency of cationic lipid includes the addition of the naphthalimide unit and [12] aneN_3_ moieties to the cationic lipids. Such a modification has led to the synthesis of functional cationic lipids with fluorescence and high photo-stability [37]. Such modifications have allowed these cationic lipids to be used in the clinical trials (Table 2). For example, Eric W F W Alton et al. reported the use of a nonviral, liposome-based formulation, GL67A, for a multi-dose clinical trial of repeated application of gene therapy in the patients of cystic fibrosis [39].

The fluorescent lipids, such as ceramide, sphingomyelin, phosphocholine, phosphatidylinositol-bisphosphate, ganglioside, cholesterol, and cholesteryl ester-based, delivery systems reported by Christian Kleusch et al. shows rapid and effective for molecular traffic analysis [41]. When the hydrophobic alkyl groups of non-polymerizable lipids are linked to pyrene and rhodamine, they are embedded into lipid bilayers of liposomes. Therefore, several fluorescent, polymerizable, metal chelating lipids have been synthesized. The pyrene-containing lipids were successfully integrated into liposomes and used as a membrane probe for lipid aggregation inside the bilayer of the liposomes. Moreover, it has been reported that in response to cupric ions, the fluorescence property of the lipid changes during the polymerization process. Further studies in this direction could be helpful in exploring the potential use of these lipids for gene delivery [42]. Moreover, didodecyldimethylammonium bromide (DDAB) has been reported to penetrate the thicker biological specimens with rapid fluorescence making it a useful tool for fluorescence imaging in living cells without any advert cytotoxicity and acceptable cell membrane permeability [43].

#### 2.1.2. Organic Fluorescent Polymers

**Figure 4:**
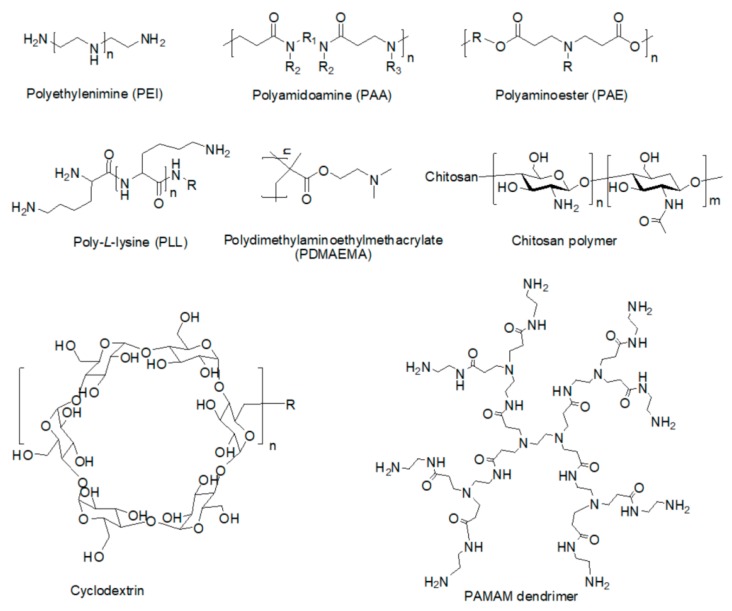
The structures of polymers.

Cationic polymers can be of natural or synthetic origin. Examples of natural cationic polymers include chitosan, cationic dextran, gelatin, cationic cellulose, and cationic cyclodextrin, whereas examples of synthetic polymers include dendrimers [1,3,10], polyethylenimine (PEI) [12], polyamidoamine (PAA), polyaminoester (PAE), Poly(ethylene oxide) and polylysine [44], and poly dimethylaminoethylmethacrylate (PDMAEMA), etc. [15]. (Figure 4).

Dendrimers are one of the structurally controlled, versatile candidates built up from branch cell monomers, which make them efficient and bio-compatible for designing gene vectors. These polymers are well-designed and involve the synthesis of low molecular weight branched precursors preceded by the systematic synthesis of high molecular weight structurally controlled dendrimers [1,3]. Polyamidoamine (PAMAM), a polycation, has also been reported as a novel siRNA delivery carrier. The interaction is based on the electrostatic interactions between the negatively charged ribose-phosphate backbone of nucleic acid and the positively charged PAMAM dendrimer. PAMAM dendrimers are modified so that they can be used as gene vectors. Such modifications include linking fluorescent compounds to the PAMAM or surface modifications. The fluorophore bound PAMAM dendrimer not only possess low cytotoxicity or high transfection efficiency but also allows the monitoring of the delivery of gene. For example, the fluorescent PAMAM dendrimer synthesized by conjugating PAMAM dendrimers to fluorescein has shown to possess low cytotoxicity, high siRNA binding affinity, with in vitro and ex vivo experiments and enhanced Cy5-siRNA delivery efficiency in A549 cells [10]. PAMAM dendrimers conjugated with the fluorescent dye Oregon green 488 are also used in combination with oligonucleotides labeled with a red (TAMRA) fluorophore for effective delivery of the antisense oligonucleotides [45]. Panagiotis Mastorakos et al. has reported the use of amine groups functionalized hydroxyl-terminated polyamidoamine (PAMAM) dendrimers to achieve effective plasmid compaction. They also have demonstrated that the use of triamcinolone acetonide (TA) can enhance nuclear localization of dendrimer-gene complex and can significantly improve cellular uptake and transfection [46]. Although high generation PAMAMs have high efficacy during gene transfection, their high cost and cytotoxicity hinder their application. Therefore, low generation amphiphilic PAMAM dendrimers have been generated by conjugating reactive oxygen species (ROS)-responsive poly(propylene sulfide) (PPS). These can condense DNA and easily release them with low cytotoxicity and high transfection efficiency [47].

Another polycationic polymer-based vector, branched polyethylenimine (PEI), has emerged as a gene delivery vector because of its high transfection efficiency. The studies have reported the modifications of PEI to synthesize fluorescent organic nanoparticles (FONs) using carbohydrates, such as glucose and sucrose. With such modifications, the resultant FONs have strong fluorescence and biocompatibility, which has made them suitable to be used in bioimaging applications, and such modifications have opened up new doors [48].

Furthermore, cationic fluorescent polymer core–shell nanoparticles are ideal polymers for the encapsulation and delivery of short interfering RNA (siRNA) as a multifunctional nanovector with good biocompatibility and high transfection efficiency [49]. Such polymers with the photoluminescent capacity synthesized from biodegradable poly (1,8-octanedio-citric acid)-*co*-polyethylene glycol are grafted with polyethyleneimine(PEI) (POCG-PEI) for delivery of siRNA and miRNA can protect them from enzymatic degradation, has low cytotoxicity, high transfection efficiency. These polymers have excellent photostability, which allows them to be used for imaging the cells and real-time tracking of the delivery [50].

#### 2.1.3. Organic Fluorescent Small Molecules

Synthetic small-molecule fluorescent compounds are powerful tools to visualize biological processes in living cells and organisms. Ever since the discovery of organic fluorescent compounds, efforts have been directed to observe the behaviors of specific biomolecules in living systems by taking the aid of these molecules as labels [51]. Such compounds include but not limited to citric acid and amine-containing and naphthalimide-based compounds. The synthesis of citric acid and amine-containing molecule-based new fluorescent dyes and polymers, which is facile and low-cost, have demonstrated that such compounds can be used in molecular labeling and cell imaging [52]. Naphthalimide-based [12] aneN_3_ compounds have been shown to have high transfection efficiency in A549 with increased transfection efficiency. Furthermore, the successful application of this vector for in situ monitoring for the mechanistic study has proved to be not only an efficient non-viral vector but also as a bio-imaging agent [8,16]. Another modification in [12] aneN_3,_ which has resulted in the synthesis of small organic non-viral gene vector, can allow fluorescent monitoring in situ, in addition to high transfection efficiency [53].

### 2.2. Metal Complexes

Metal complexes exhibit diversity in DNA binding, which results in DNA condensation. These positively charged particles attract cell membranes via electrostatic interactions, which result in entry into the cytoplasm via endocytosis. After being transported to the nucleus, the gene is transfected. Conjugating metal complexes with biomolecules and nanomaterials have been an emerging interest not only for molecular imaging but also for anticancer therapy. In the beginning, nanomaterials were employed as delivery carriers to guard the drug or imaging agents from degradation [54].

Because of the increasing need to introduce a large-sized gene into the cell, the metal complexes have been employed as DNA condensing agents. Cationic metal complexes, such as hexammine cobalt(III) cation (Co(NH_3_)_6_)^3+^, have been reported to induce DNA condensation and numerous other metal complexes, such as Co(III), Co(II), Fe(II), Ca(II), Cu(II), Ni(II), Zn(II), Ru(II), Pt(II), and La(III), have also been proved to be DNA condensing agents, that are nonreactive to DNA. The potentiality of dinuclear Cu^2+^ and Co^2+^ complexes of polybenzimidazole ligands has been employed as DNA carriers to express the internalized DNA [5,25,26]. A series of metal complexes with polybenzimidazole (pbzim) can help the internalize DNA condensate into the cells with high cell transfection and low cell toxicity, suggesting their potential as a new and possible non-viral carrier for DNA delivery [5,27].

Apart from their use as a DNA condensation agent, the metal complexes are used to help track the gene delivery. The use of fluorescence is the efficient mean for tracking gene delivery in living cells, and the transition-metals, such as tetranuclear ruthenium(II), has been reported to show fluorescence. Lipophilic tetranuclear ruthenium(II) complexes synthesized for gene delivery not only can condense DNA but also help to track DNA movement in living cells [55]. Despite the use as a DNA condensation agent and gene delivery tracker, the use of biopolymers as gene vectors raise difficulty when their ability to transfect and tolerance to serum degradation is taken into consideration. The conjugation of Zn(II) to polycations derived from cyclen reported by Qing-Ying Yu et al. can induce apparent conformational changes of DNA and better DNA release, which might favor the gene transfection process. In addition, the transfection efficiency of these complexes can be increased by coordinating Zn(II), and these complexes can tolerate serum degradation better than polyethylenimine (PEI) [56].

### 2.3. Quantum Dots

Though there have been successes in the synthesis of siRNA delivery methods using tracking agents, their use was limited due to the loss of fluorescent signals. Hence, designing not only an efficient but also self-tracking delivery system has become a principal research area. Quantum dots (QD), therefore, contribute ‘their share’ in the development [24]. Quantum dots are nanometer-sized semiconductor particles with advantages over traditional organic dyes on several counts, one of the most obvious being brightness as well as their stability [57].

As the traditional QDs are produced from heavy-metal elements, such as PbSe, CdS, and CdSe, which results in poor biocompatibility and high toxicity, photoluminescent carbon quantum dots (CQDs) are gathering considerable attention due to their excellent biocompatibility, low toxicity, strong fluorescence, etc. Because of the surface modifications, such as the addition of cation and peptides on QDs, the development of the new generation of systems with additional features has become possible.

Optically tunable carbon quantum dots (CQDs) synthesized through one-step hydrothermal carbonization have shown a strong capacity of plasmid DNA condensation, fine biocompatibility, low toxicity, and high transfection efficiency and the ability to track their own paths of entering into the cells [21].

Biocompatible nanocomposites consisting of quantum dots, such as orange fluorescent Mn^2+^-doped ZnS QDs, could act as an efficient probe. Moreover, coating QDs with chitosan, a naturally available biopolymer, can easily lower the QD toxicity due to its biocompatibility [22]. The development of the carrier by combining water-soluble quantum dots and plasmid DNA by obtaining QDs with different charges and then by conjugating plasmid DNA to QD has shown to enhance resistance to nucleases digestion and successful penetration of cell membrane, high transfection efficiency, and low cytotoxicity than the commercial liposome transfection vector [23]. The excellent fluorescent properties and downregulated expression of the gene in oral squamous cell carcinoma Tca8113 cells by application of cationically-modified QDs to deliver siRNA only prove their capabilities as non-viral vectors [24]. Another modification, i.e., PEGlyated core to the QD and addition of siRNA and tumor-homing peptides (F3) on the surface, has enabled the efficient delivery of F3/siRNA-QDs to HeLa cells [29].

### 2.4. Gold Nanoparticles

**Figure 5:**
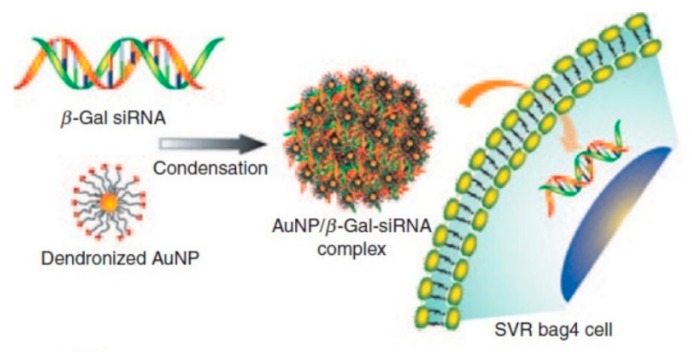
Schematic illustration of G2-AuNP/β-gal-siRNA complexation and transfection into SVR-bag4 cells [32].

Nanoparticle-mediated transfection is defined by low immunogenicity and a low production cost, as well as an easy synthesis process compared to other viral delivery systems [30]. Inorganic nanoparticles (NPs), including gold [30,32] and silica [29], are some of the promising non-viral gene delivery vectors that have been used to deliver drugs. Gold nanoparticles are known to be non-cytotoxic, biocompatible, tunable size, and straightforward functionalization, which make them suitable for the synthesis of nucleic acid delivery carriers (Figure 5) [29,32]. However, many of them are insoluble and easy to aggregate in biological media, which limits their biomedical applications. Therefore, a wide range of encapsulation methods has been developed to enhance their solubility in aqueous solutions and prevent aggregation. Gold nanoparticles encapsulated in graphene oxide (GO) can decrease the cytotoxicity and upon adding attachments for polyetheyleimine (PEI), exhibit high transfection efficiency while retaining 90% viability of HeLa cell [58].

Due to polydispersity, the formulation of stable and single dispersed nanoparticles is a challenging task. But the addition of charged molecules on the surface can be a new direction in solving these problems [59]. The other modifications in gold nanoparticles have led to the generation of more effective vectors. Such modifications involve PEGylation, layering of charge-reversal polyelectrolyte, coating with lysine-based headgroups, etc. PEGylation of gold nanoparticles reduces the PEI cytotoxicity and also enhances nanoparticle dispersion in culture media. The different plasmids with a different number of pair bases can be used for transfection using this vector, and plasmids of up to 40 kbp have shown a superior performance when transfected with this vector, compared to commercially available lipoplexes [30]. High fluorescent Ag–Au–PEI conjugates showed high transfection efficiency and low cytotoxicity toward B16F10, HeLa, and CHO cells. Most importantly, a high quantum yield of 14.56% made this system not only a useful probe for intracellular trafficking but also in vivo bioimaging by using BALB/C nude mice [31]. In addition, the charge-reversal polyelectrolyte deposition on gold shows effective enhanced gene delivery efficiency with regulated expression of the gene under acidic environment. This could be due to gold nanoparticle/nucleic acid complexes escape from endosome/lysosome facilitated by such layering resulting in the release of functional nucleic acids into the cytoplasm [60]. Coating gold nanoparticles with lysine-based headgroups produces active transfection vectors without any observable cytotoxicity. Moreover, these nanoparticles could also be used for controlled release and parallel DNA expression due to their ability to respond to intracellular glutathione levels [61].

## 3. The Targeting Vectors

### 3.1. Nuclear Targeting Non-Viral Vectors

The transfer of DNA into the cell nucleus remains the key step for gene delivery and expression. SV40 DNA targeting sequences (DTS) are capable of stimulating nuclear entry by binding of transcription factors having nuclear localization signals, thereby coating the plasmid DNA and directing nuclear entry of the non-viral plasmid-based vector.

The first prototype skeletal targeting vector with the chimeric hRunx2-hCo1Ia2 promoter can direct in vivo Ob-specific transgene expression [62]. By using recombinant DNA technology, a multi-functional gene delivery system for nuclear targeting has been designed. The example of such a delivery system is spider dragline silk recombinant proteins, altered with DNA condensing units utilizing proton sponge endosomal escape pathways for the enhanced delivery. This delivery system can enhance short-term transfection efficiency in a COS-7 cell line (adherent kidney cells isolated from African green monkey) compared to lipofectamine 2000 and polyethyleneimine (PEI), as was cell viability [63].

**Figure 6:**
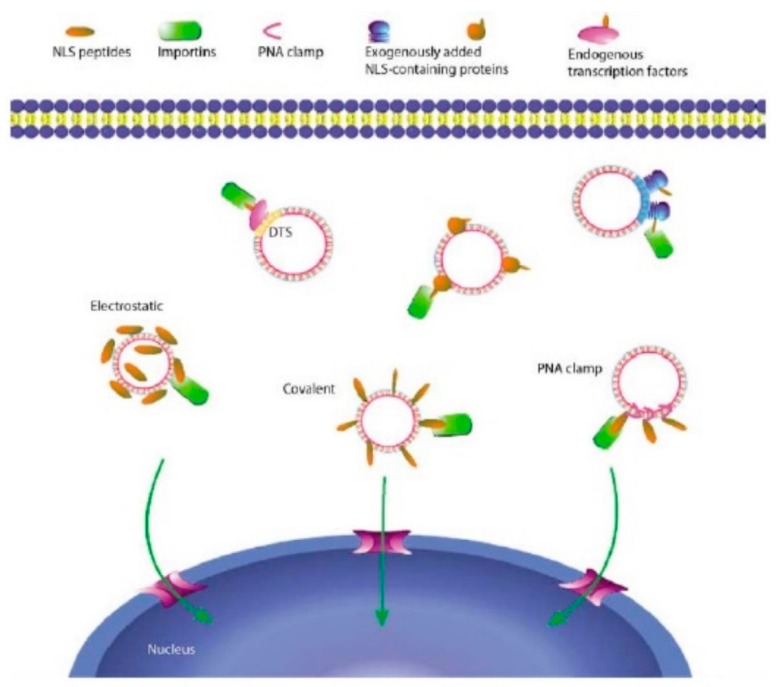
Various methods to enhance nuclear localization [64] (Reprinted by permission from Springer Nature and Copyright Clearance Center: Springer Nature, Gene Therapy, Nuclear entry of nonviral vectors, D A Dean et al. 2005.).

During the gene transfer process, many cells that are of target interest do not actively undergo cell division. Therefore, nuclear transport in the non-dividing cells is of significant importance. One of the methods proposed to enhance the nuclear import relies on the linking of NLS-peptides or NLS-containing proteins by either electrostatic, covalent, or PNA clamps to the DNA (Figure 6) [64].

Nuclear localization sequence (NLS) peptides linked to DNA, by creating a union between the tetracycline repressor protein (TetR) and the SV40-derived NLS peptide, can increase the accumulation of fluorescence-labeled DNA after TetR-NLS binding in the nucleus confirming intracellular trafficking [65]. The fluorescent carbon nanospheres can also be used as a nuclear targeting vector to induce the hyperacetylation of histone acetyltransferase (HAT) p300 as well as histones both in vitro and in vivo with localization in the brain, the liver, and the spleen of the mice. Moreover, the activation of HAT-dependent transcription has been shown to establish an alternative course for the activation of gene expression mediated by the induction of HAT activity instead of histone deacetylase (HDAC) inhibition [66].

### 3.2. Mitochondrial Targeting Non-Viral Vectors

Mutations in mitochondrial DNA are a recurrent cause of metabolic and neuromuscular abnormalities and appear to have associated with Parkinson’s, Alzheimer’s diseases [67], and myocardial ischemia-reperfusion injury (MI/RI) [68]. Molecules housed on or inside the mitochondria are considered key targets, and an extensive range of research has undertaken to develop targeted therapies to target the molecules causing various diseases [69]. But due to the negative potential and special bilayer structure of the mitochondria, the delivery of the therapeutic molecules to mitochondria has become difficult.

**Figure 7:**
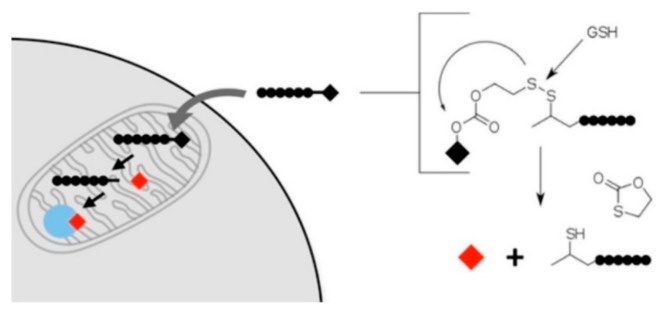
Linker mediated mitochondrial transport [72].

To prevail over these barriers, the researchers have developed a range of preparations, such as liposomes, polymeric nanoparticles, triphenylphosphonium (TPP), etc. [70]. Iridium (Ir)-loaded PEGylated liposomes (Lipo-Ir) can maintain sustained release, exceptional biocompatibility, and physical stability. The MTT assay showed that Lipo-Ir and Ir can lead to high cytotoxicity against selected cancer cells [71]. The application of fluorescent compounds and amphiphilic cation in preparation of mitochondrial targeting vectors has shown to improve not only internalization but also transfection efficiency. Such as the use of rhodamine plasmid DNA nanoparticles with suitable size and morphology have shown the promising new way towards mitochondrial gene therapy. The internalization of such nanoparticles into the cell was observed by fluorescence microscopy, while the confirmation of delivery was achieved through fluorescence confocal microscopy [67]. The nanoparticles synthesized by conjugating PLGA to PEG and SS31 peptide to treat MI/RI can deliver cyclosporine A into the mitochondria of MI/RI rats without any overt cytotoxicity [68]. The use of triphenylphosphonium (TPP) in preparation of mitochondria targeting vectors, such as TPP conjugated dendrimers, have shown that these vectors can target mitochondria with efficient transfection efficacy in addition to low cytotoxicity and higher efficacy [3]. The covalent linking of small molecules to mitochondria-penetrating peptides (MPPs) MPPs can adversely affect the activity of the load against its cellular target. Therefore, the cleavable linkers have developed for the easy release of chemical cargo from following mitochondrial transport (Figure 7) [72].

### 3.3. Tumor Targeting Non-Viral Vectors

#### 3.3.1. Folate-Linked Nanoparticles

The majority of cancer tissues overexpress folate receptor (FR) to which folate binds, and this ability and affinity have been exploited to induce folate-conjugated nanoparticles to bind and enter cancer cells [73,74]. Much research has been undertaken so far to develop different vectors, such as a folate-linked lipid-based nanoparticle, mPPS-FA (methyl PEG-PEI-sebacoyl chloride-Folic acid), etc. Folate-linked lipid-based nanoparticles used to deliver an NFk B decoy into activated murine macrophage-like RAW264.7 cells have shown an inhibitory effect on the translocation of NFk B into the nucleus [75].

The study conducted by Takashi Yoshizawa et al. has shown that folate-linked nanoparticles can deliver synthetic siRNA into nasopharyngeal tumor KB cells with high transfection efficiency and selectivity. In vivo experiments showed significantly suppressed KB xenografts growth in mice xenografted with KB cells proving their potential as an effective vector in nasopharyngeal cancer synthetic siRNA gene therapy [76]. A gene delivery vector, mPPS-FA, synthesized by conjugating folic acid to a backbone produced by coupling methyl PEG-2000, PEI-600, and sebacoyl chloride showed >80% cell viability indicating low cytotoxicity and high GFP transfection efficiency [77]. Folate-linked nanoparticles (NP-F) developed for human prostate cancer LNCaP and PC-3 cells and nasopharyngeal cancer KB cells suicide gene therapy has shown high DNA transfection efficiency in KB, LNCaP, and PC-3 cells as well as cell growth inhibition in LNCaP cells in the presence of ganciclovir (GCV) enhanced with *HSV-tk* and *Cx43* genes in male BALB/c nu/nu mice with KB tumor xenograft [78].

#### 3.3.2. Galactose Modified Non-Viral Vectors

Sugar-macromolecule conjugates are another exciting approach among a developing array of medicinal applications for carbohydrates as selective drug delivery systems [79]. The interaction between *β*-*D*-galactose-functionalized water-soluble pillar[5]arene (GalP5) and camptothecin prodrug (G) containing a disulfide bond has shown to produce GSH-responsive supramolecular prodrug nanoparticles. The resultant prodrug nanoparticles have reported being stable under physiological conditions. Moreover, in vitro studies have demonstrated the entry of these prodrug nanoparticles into HepG2 cells overexpressing asialoglycoprotein receptors has shown maximum anticancer efficacy and reduced side effects [80]. While a series of novel cationic glycolipids containing sugar heads reported for selective targeting of genes to *Balb/c* mice liver can transfect gene efficiently, for this efficient gene transfection, cationic glycolipids with cyclic sugar-head require longer spacer arms than their acyclic sugar-head counterparts [81].

Galactose or *N*-acetylgalactosamine is known to be recognized and incorporated into the cells through the asialoglycoprotein receptor (ASGPR) that is exclusively expressed on hepatocyte and hepatoma. The synthesis of a galactose-modified lipid with an aromatic ring with click chemistry and complex with DNA termed as lipoplex has an ability to interact with ASGPR immobilized on gold substrate in the quartz-crystal microbalance (QCM) sensor cell and to induce high gene expression in HepG2 cells but not in A549 cells [82]. Novel co-polymer LA-PegPI, having low-molecular-weight polyethylenimine (PEI) cross-linked by myo-inositol (INO) and complexed with a galactose-grafted PEG chain, exhibits excellent stability in physiological conditions, low cytotoxicity, and high transfection efficiency in the asialoglycoprotein receptor (ASGPR)-positive liver cells in vitro. The intraperitoneal injection of LA-PegPI/pIL15 nanoparticles resulted in effective suppression of tumor growth and prolonged survival time of hepatocellular carcinoma tumor-bearing *Balb/c* mice by activation of CD8^+^ T cells and NK cells and upregulation of the cytokines with effective stimulation in the proliferation of NK cells [83].

### 3.4. Bone Targeting Non-Viral Vectors

The primary cancer of the breast, lung, and kidney has a powerful capacity of metastasis and/or invasion to the bone leading to osteosarcoma [84]. Because of the poor prognosis, it has a high degree of malignancy. To improve the concentration of anti-tumor drugs in the bone tumor tissue and decrease the drug’s toxicity, the bone targeting systems have been a major focus of many studies [85,86]. The bone-specific delivery of proteins having aminobisphosphonate (aminoBP) conjugated to bovine serum albumin (BSA) exhibit a high affinity to bone in vitro and in vivo with enhanced bone delivery of BSA by 2.2-fold to 7.5-fold [87]. Conjugates of bisphosphonates and prostaglandin E2 also have the ability to bind to bone and support bone formation by the local enzymatic release of prostaglandin E2 from a conjugate compound [88].

**Figure 8:**
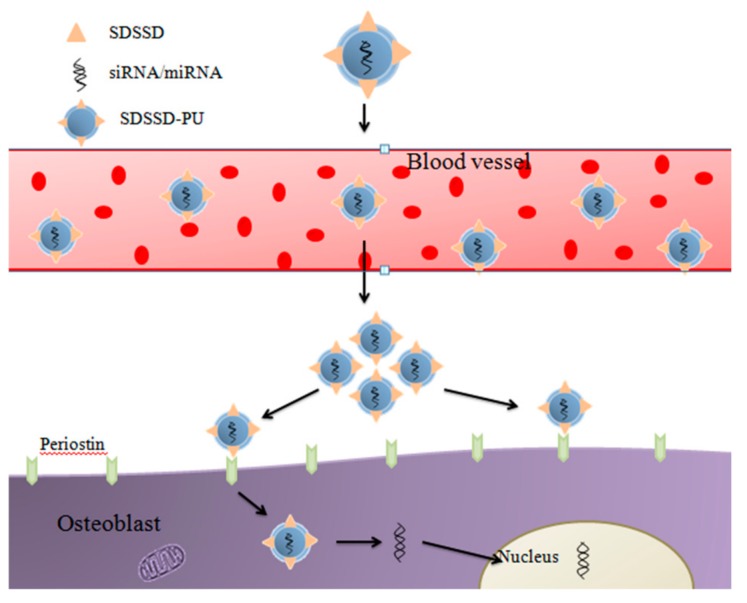
Schematic of SDSSD based delivery system.

Recently, the research has oriented in the development of targeted systems to target either osteoblast [89] or bone formation surfaces [90] or bone resorption surfaces [91]. The system developed by Yao Sun et al. employs the osteoblasts targeting peptide-based Ser-Asp-Ser-Ser-Asp (SDSSD)-modified polyurethane (PU) nanomicelles (Figure 8). These nanomicelles encapsulating anti-miRNAs for delivery to osteoblasts had shown the selective targeting abilities of nanomicelles not only to osteoblasts but also to the bone formation surfaces without explicit toxicity or bringing out any immune response in vivo. Furthermore, results showed increased bone formation, bone micro-architecture, and bone mass in an ovariectomized osteoporosis mouse model [89].

While Ge Zhang et al. reported dioleoyl trimethylammonium propane (DOTAP)-based cationic liposomes linked to six repetitive sequences of aspartate, serine, serine ((AspSerSer)_6_) as a targeting moiety for bone formation surfaces. The encapsulation of an osteogenic siRNA using this system has reported its delivery to bone formation surfaces [90]. A targeting system developed by linking *D*-Asp_8_ peptide to liposome for delivering antagomir-148a can facilitate the enhanced antagomir-148a with the downregulation of miR-148a in osteoclasts in vivo. The results have shown reduced bone resorption and less intense deterioration of trabecular architecture in osteoporotic mice [91].

## 4. Biodegradable Non-Viral Vector

As the research on non-viral vectors is progressing, the materials that are being used, though they give promising results towards transfection and low cytotoxicity, raise the question of their biodegradability. Many vectors employ the use of metal ions and polymers for synthesis, and their compatibility and ability to undergo biodegradation remains a problem. Therefore, the synthesis of biodegradable non-viral vectors has become an important aspect in the development of non-viral vectors.

In recent years many biodegradable polymers not only have been synthesized but also have been modified to deliver the desired compounds into the cells, such as poly(amino esters), nanoparticles, etc. The synthesis of a new class of biodegradable poly(amino ester) compounds synthesized by Qiang Liu et al. can give higher transfection efficiency and lower cytotoxicity in HEK 293 cells, due to their biodegradable backbones and hydroxyl groups [92]. Whereas, encapsulation of PEI/siRNA polyplexes encased within oppositely charged biodegradable and biocompatible polyelectrolytes polymer capsules show stability and high in vitro transfection efficiencies of siRNA by gene silencing and reduced PEI-derived toxicity which demonstrates their biodegradability with efficiency in delivering and transfecting nucleic acids [93]. Moreover, the co-assembly of PEI with other compounds has shown to reduce the cytotoxicity of PEI. Such a co-assembly of PEI-CG-PEI or co-assembly with PEG-CG reported by Weiwei Wang et al. for DNA and siRNA delivery have demonstrated the condensation efficiency, glycolide induced degradability in addition to decreased cytotoxicity and improved stability of PEI in the serum because of the shielding effect by PEG [94].

Because of their small size and easy accessibility and biodegradability, nanoparticles are used for efficient delivery of siRNA. The study reported by Jing Du et al. demonstrated that NPs loading Cy3-labeled siRNA had much higher intracellular siRNA delivery efficiency and gene silencing efficiency in SPC-A1-GFP cells [95]. Whereas, lipopolymers (LPs) have the ability to condensate plasmid DNA to form nanoparticles. Furthermore, it has revealed that lipopolymer had much higher transfection under the polymer/DNA weight ratio of 0.8 in A549 cells [96]. Based upon low generation (G1) peptide dendrimer, cationic polymers with disulfide-containing linkages has been synthesized by Chun-Yan Li et al. The bioreducible property of these polymers has been studied by using dithiothreitol (DTT). Furthermore, these materials have the ability to condense the DNA into nanoparticles and exhibit much higher gene transfection efficiency and reduced cytotoxicity in both HEK293 and U2OS cells [97].

Another multifunctional, amphiphilic biodegradable cationic triblock copolymers synthesized based on poly(ethylene glycol), poly-ε-caprolactone, and polyethyleneimine have shown the capability to deliver siRNA in vivo as well as in vitro. The hydrophobic copolymer can facilitate high cellular uptake with good knockdown efficiency in the lungs of six-week-old BALB/c mice [98]. Cyclen-based linear and cross-linked polymers having biodegradable ester or disulfide bonds have good DNA-binding and plasmid DNA retardation abilities. In addition, MTT assay of cross-linked polymers has indicated low cytotoxicity toward A549 and 293 cells [99]. To increase cellular uptake and endosomal escape efficiency, a non-viral gene has been reported to deliver plasmid DNA into the cell. The results indicated high cellular uptake, endosomal escape, and improved transfection efficiency in human cervical carcinoma (HeLa), rat cardiomyocytes (H9C2), and colon carcinoma (CT26) cells [100]. Other modified PEI biodegradable copolymers, such as diblock copolymers (MPEG-*b*-PCLs), have been stated to exhibit a low toxicity profile in addition to a significant enhancement of transfection activity in comparison to the low MW PEI [101]. Such diblock copolymers (MPEG-*b*-PCLs) synthesized from poly(ϵ-caprolactone) (PCL) and monomethoxyl poly-(ethylene glycol) (MPEG) reported by Xintao Shuai et al. have lower toxicity of copolymer-based complexes, good biocompatibility, potential biodegradability, and comparatively high gene transfection efficiency [102].

Exosomes are extracellular nano-sized vesicles released by most cells but not all, emerging as a promising tool for therapeutic delivery. They intercede intercellular communication via the transfer of genetic information to recipient cells by crossing biological membranes facilitating the delivery of their cargo to their targets. They are stable, biocompatible, capable of stealth when circulating in the bloodstream, and able to overcome natural barriers. Given these natural properties, the opportunity to utilize these vesicles for therapeutic purposes is being explored [103,104,105]. The extracellular vesicles (EVs) released in the brain modulate the cross-talk between neurons, astrocytes, microglia, and oligodendrocytes, in central nervous system (CNS) physiology, in neurodegenerative and neuroinflammatory disease states as well as in glioma [104]. As they can carry DNA, RNA, proteins, doxorubicin, curcumin, or paclitaxel and can be employed as effective and non-immunogenic drug carriers [106], EVs are being mainly studied in the field of cancer, but are also increasingly examined in immune-related diseases and regenerative medicine [107]. Conversely, due to a lack of cell-targeting specificity, their use as a therapeutic agent is limited. To improve the specificity, EVs are modified with targeting ligands, such as polyethylene glycol (PEG), leading to extended circulation time and increasing EV accumulation in targeted tissues and improving cargo delivery [108].

## 5. Other Functional Vectors

### 5.1. Carbon Nanotubes Based Functional Vectors

Carbon nanotubes (CNTs) are the carbon allotropes with a cylindrical nanostructure. Because of their many capabilities, such as extraordinary thermal conductivity and electrical and mechanical properties with strength and flexibility, showing strong optical absorbance in the NIR region, these have been used in drug delivery and photothermal therapy [29,109]. A drug delivery system synthesized by PEG conjugation to formulate stable and efficient CNTs for the anti-cancer drugs doxorubicin and mitoxantrone has shown stability under biological conditions, sustained release, and promoted selectivity. The pH-dependent drug release in buffer solutions and the cellular uptake of nanotubes with intracellular drug release has confirmed by confocal microscopy. Such targeted drug-loaded nanotubes allow for sustained release, which reduces drug-related side effects in vivo and in vitro [110].

To permit the development of a complex between carbon nanotubes with DNA, the surface modifications can be made. These involve multi-walled carbon nanotubes (MWCNTs) modification to enhance transfection, CNTs length reduction, and attachment through electrostatic interactions, etc. [111]. Single-walled carbon nanotubes (SWCNTs), a type of carbon nanotube, in conjunction with fluorescent dyes, such as acridine orange (AO), have been in use to monitor the location of SWCNTs, which emit green fluorescence in an acidic environment [112]. Due to poor solubility, CNTs are poorly soluble in water. Therefore, many studies have been involved to increase the solubility of CNTs. Many compounds, such as carnosine, with CNTs complexes, are used to test the solubility. The computational study conducted by Sepideh Ketabi and Leila Rahmani on the solubility of carnosine-CNTs showed that the functionalization of CNT with carnosine dipeptide significantly improves solubility in biological fluids and their biocompatibility [113]. Lysosomal escape is an important aspect of designing the effective transfection vector. Double-walled carbon nanotubes (DWCNTs) modified by coating polyethyleneimine has produced an efficient vector not only for gene delivery to human cells but also for drug delivery by escaping lysosomal degradation. It also reported as having shown no cytotoxicity against the cells [114]. Artificially produced organic polymers are the most routinely used materials for surface modifications of CNTs. Because of their cost and inability to undergo biodegradation, natural biopolymers have been in use, such as chitosan. The multi-walled CNTs (MWCNTs) of different length functionalized with chitosan–folic acid nanoparticles have an ability to deliver the plasmid DNA of enhanced green fluorescent protein (pEGFP-N1) into HeLa and MCF-7 cells in addition to decreased cytotoxicity as well [115]. The significant therapeutic efficacy of CNT-mediated siRNA delivery has an exception in vivo. Therefore, this will be where the future exploitations of CNT-mediated gene therapy can be undertaken [2].

### 5.2. Graphene-Based Functional Vectors

Carbon nanomaterials, such as graphene (G) and graphene oxide (GO), have advanced biomedical applications. Graphene is two dimensional (2-D) single or few layers of sp^2^-bonded carbon atoms arranged in a honeycomb. Graphene is a basic building block for other graphitic materials, such as zero-dimensional fullerenes, carbon nanotubes, CNTs, graphite, etc. Because of its excellent physical, chemical and mechanical properties, it has evoked enormous interest [116,117,118]. It has been shown that when GO complexed with cell-penetrating peptides (CPPs) and plasmid (pGL3), splice correction oligonucleotides (SCO), or small interfering RNA (siRNA), it lowers the cytotoxicity of CPPs and improves the material biocompatibility as well as increases the cell transfection [117].

**Figure 9:**
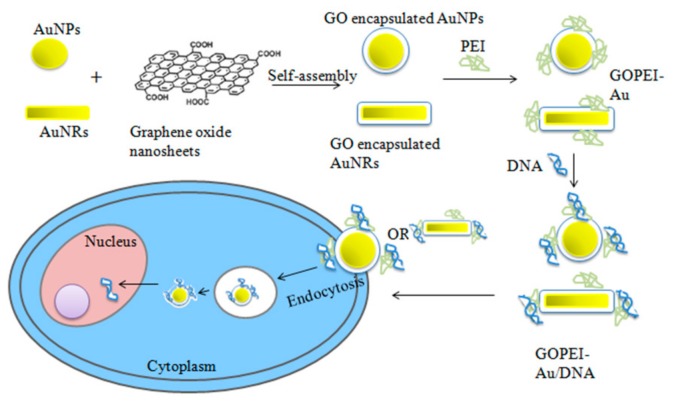
Schematic illustration for the synthesis of GOPEI-AuNPs and GOPEI-AuNRs and the possible mechanism of gene delivery using GOPEI-AuNPs as a carrier.

In various biomedical fields, graphene and its derivatives, as carbonic nanomaterials, are exploited because of their biocompatibility as nano-carriers. The surface modifications have allowed researchers to improve biostability, cellular uptake, and increase gene loading capacity by conjugating various polymers or ligands [119]. GO nanosheets can be used to encapsulate nanoparticles, such as gold and nanorods (Figure 9). When complexed and used for transfection to HeLa cells, they exhibited good DNA binding ability and condensed plasmid DNA into nanoscale particles (150 nm). In vitro gene transfection demonstrated much lower cytotoxicity and higher efficiency [58]. GO assembled nano-aggregates cross-linked by ATP-responsive DNA strands developed for ATP-mediated controlled drug release system with high drug loading capacity, and ability of site-specific drug release has made it a promising aid for enhanced therapeutic efficacy in cancer treatment [120]. Due to the low cytotoxicity of graphene, its surface modification has led to the construction of new gene delivery systems. Such modifications may include conjugation with alkylated derivatives of different cationic polymers, such as polyethylenimine (PEI), polypropylenimine (PPI), and polyamidoamine (PAMAM), by linkers, such as surface carboxyl group, glycine, and spermidine. The transfection efficiency of these vectors, when evaluated by using green fluorescent protein (GFP) plasmid, PEI–GO conjugate bearing glycine linker, was found to be the most efficient vector [121]. It has also been demonstrated by enhanced green fluorescent protein (EGFP) transfection in HeLa cells that GO with polyethylenimine (PEI) is able to deliver genes inside the cells with mitigated cytotoxicity and improved transfection efficiency [116].

### 5.3. Magnetic Nanoparticles (MNPs)

To lower the side effects of traditional cancer therapy and diagnosis, significant research efforts are devoted to the finding of efficient approaches, and magnetic nanoparticles have proved to be one of them. Magnetic nanoparticles have attracted much attention because of their unique physical properties, magnetic susceptibility, biocompatibility, stability, and have been used for disease imaging via passive targeting [122,123].

Metal or metal oxide nanoparticles, such as superparamagnetic iron oxide nanoparticles (SPIONs), are the most commonly used materials for magnetic drug delivery. Iron oxides with core/shell structure, such as hematite, maghemite, magnetite, and some others, have been investigated, but only maghemite and magnetite have been found to be of greatest interest [124,125]. The report by Michele K.Lima-Tenório on MNPs showed great potential, and under suitable application and oscillation of an external magnetic field, the magnetic energy of the particles is converted to thermal energy, thereby killing the cancer cells in cancer cells (U87MG) xenografted nude mice [122].

The investigation of iron oxide nanoparticles (IONPs) for delivery of therapeutic DNAzyme for the treatment of hepatitis has demonstrated efficiency to induce knockdown of the hepatitis C virus gene, NS3, required for the virus replication [29]. Iron oxide nanocrystals coated with poly(succinimide) grafted with folate-conjugated polyethylene glycol (PEG) and alkyl chains can be used to target and detect cancer cells. Even at high concentrations, these magnetic nanoparticles displayed low cytotoxicity and exhibited highly efficient intracellular uptake into KB cells. Within 3 h, the mouse bearing a KB cell tumor displayed a 75% drop in the T_2_ signal in the tumor tissues shown by in vivo magnetic resonance (MR) images. These results indicate the accumulation of these MNPs at the tumor site and their effectiveness in the detection of a tumor using in vivo MRI methods [73]. These magnetic nanoparticles have also approved by US-FDA for treatment for a variety of diseases. Iron oxide under the trade name of Nanotherm has also been approved by US-FDA for Glioblastoma in 2010. Iron dextran with low molecular weight (MW) and high MW has been approved for iron deficiency in chronic kidney disease (CKD) in 1957 to allow an increased dose. SPIONs coated with dextran and silicones are also approved by US-FDA as an imaging agent in1996 and 2008, respectively [126].

### 5.4. RGD Modified Polymers

Arginylglycylaspartic acid (RGD) is a peptide motif responsible for cell adhesion to the extracellular matrix (ECM). Integrins recognize and bind to this sequence [127]. Arginine-glycine-aspartic acid (RGD) peptide shows the excellent specific binding ability for α_ν_β_3_ integrin, and RGD-modified polymers have been studied in the field of drug delivery system for chemotherapy, and many outstanding results have been achieved, showing not only that RGD-modified polymers have a broad application but also enormous development prospects [128].

Gold surfaces modified with a thiol-functionalized arginine-glycine-aspartic acid (RGD) peptide used for examination of cell adhesion and detachment were able to keep cells of interest living and intact during experiments, making it possible to quantify cell adhesion and detachment [129]. A fluorophore-tagged RGD peptide designed to control endothelial cell adhesion revealed that partial RGD surface coverage is sufficient to create integrin signaling leading significantly increased cell spreading and focal adhesion kinases (FAK) activation, as well as allowing primary endothelial cell expansion in serum-free medium and real-time monitoring of micropattern effects on endothelial cells [130]. In addition, RGD modified natural polymers, such as collagen and hyaluronic acid, have shown promising results for medical applications, such as implantation into the lesioned optic tract or cerebral cortex of rat brains, and to study their ability to promote regeneration of bone in a rabbit model [131].

## 6. Future Directions and Concluding Remarks

In the past decades, considerable progress has been achieved in different areas associated with gene-based therapy, including the improved effectiveness and stability of nucleic acids as well as the development of new delivery materials. Due to advancements in genomics and structural biology, our understanding of the genetic basis of disease has greatly enhanced and has provided a wide array of new targets for genetic medications. Therefore, the development of viral and non-viral gene vectors has been the area of research of any investigators. As compared to viral gene vectors, non-viral gene vectors have shown promising results with transfection, biocompatibility, safety, stability, etc.

These advantages have led to increasing research in the designing of novel delivery systems with different modifications that have enabled researchers to deliver desired therapeutic agents to the desired site to treat the diseases. The non-viral vectors that show reduced transfection efficiency or relatively high cytotoxicity have opened the new doors for the improvement leading to the development of functional fluorescent and non-fluorescent vectors, such as organic fluorescent vectors, quantum dots, metal complexes, and fluorescent nanoparticles, non-fluorescent vectors include targeting vectors, biodegradable vectors, carbon nanotubes, graphene-based vectors, and magnetic nanoparticles. These vectors not only carry the nucleic acid to the desired sites but also due to their fluorescent properties, they help to track the progress of the vectors inside the cell.

Though these vectors show biocompatibility, stability, the mechanism of gene transfection, low transfection efficiency, and mechanism of host response remain major concerns. Though there is work that reports that by modifying the vector, one can achieve high transfection efficiency, more research in this area is needed to make the systems suitable for in vivo use. The research in these areas may significantly improve our understanding of gene transfection, host response mechanisms and provide important insights for the development and improvement in other non-viral gene vectors for targeted nucleic acid delivery and novel drugs against cancer and other major genetic diseases.

## Figures and Tables

**Table 1:** Advantages and disadvantages of viral vectors [9].

Vectors	Virus	Advantages	Disadvantages
Viral	Retrovirus	Long-term gene expression	Low efficiency in vivo, immunogenic problems, the inability to transduce the nondividing cells, and the risk of insertion, infects dividing cells only.
	Lentivirus	Long-term gene expression, infects non-dividing and dividing cells	Generation of replication-competent virus, Potential for tumorigenesis
	Vaccinia virus	High immunogenicity safety: used as a smallpox vaccine, high titer production	Pre-existing immunity
	Adenovirus	Transfect dividing and non-dividing cells and have low host specificity, deliver large DNA particles (up to 38 kb), high immunogenicity safety: used in many clinic trails, high titer production	Gene expression is too short term, pre-existing immunity.
	Adeno-associated virus	Long-term gene expression, non-pathogenic virus	Low titer production
	Cytomegalovirus	Induces a unique CTL response, protects against SIV infection in an animal model	Pre-existing immunity risk of pathogenesis in specific individuals

**Table 2:** Liposome-mediated clinical trials for cancer gene therapy [40].

Cancer	Major Carrier	Gene	Administration Route	Phase (Start Year)	Note
Stage IV melanoma	DC-Chol	HLA-B7	Intratumoral, Intrapulmonaty	Phase I (1993)	
Head and neck cancer	DC-Chol	EGFR antisense	Intratumoral	Phase I (1999)	
Head and neck cancer, breast cancer,	DC-Chol	E1A	Intratumoral with catheter	Phase I (1999)	
Breast cancer, ovarian cancer	DC-Chol	E1A	Intrapleural, Intraperitoneal	Phase I (1999)	
Ovarian cancer	DC-Chol	E1A	Intraperitoneal	Phase I/II (2004)	tgDCC-E1A in combination with paclitaxel
Head and neck cancer	DC-Chol	E1A	Intratumoral	Phase II (2002)	tgDCC-E1A
Metastatic melanoma	DMRIE	HLA-B7/β2-microglobulin	Intratumoral	Phase I (1997)	
Metastatic melanoma	DMRIE	HLA-B7/β2-microglobulin	Intratumoral	Phase II (2002)	Allovectin-7 alone
Stage 3 or Stage 4 melanoma	DMRIE	HLA-B7/β2-micrOglobulin	Intratumoral	Phase III (2006)	Allovectin-7 alone compared with chemotherapy
Head and neck cancer	DMRIE	HLA-B7	Intratumoral	Phase I (2001) Phase II 2002)	Allovectin-7
Prostate cancer	DMRIE	IL-2	Intraprostatiscal	Phase I/II (2000)	Leuvectin
Leukemia	DOTIM	Non-coding plasmid DNA	Vaccination	Phase I (2009)	As an adjuvant (JVRS-100)
Advanced solid tumor, advanced malignancy	Cationic cardiolipin	c-raf antisense	Intravenous	Phase I (2004)	LErafAON-ETU
Refractory or Relapsed Acute Myeloid Leukemia, Acute Lymphoblastic Leukemia, and Myelodysplastic Syndrome	Unknown	L-Grb-2 antisense	Intravenous	Phase I (2010)	

DC-Chol-: 3beta-[N-(N′,N′-Dimethylaminoethane)-carbamoyl]cholesterol, DMRIE-: 1,2-dimyristyloxy-propyl-3-dimethyl-hydroxy ethyl ammonium bromide, DOTIM-: 1-[2-(oleoyloxy)ethyl]-2-oleyl-3-(2-hydroxyethyl)imidazolinium chloride.
